# RETRACTED: Atta et al. Synthesis and Application of Hybrid Polymer Composites Based on Silver Nanoparticles as Corrosion Protection for Line Pipe Steel. *Molecules* 2014, *19*, 6246–6262

**DOI:** 10.3390/molecules31142470

**Published:** 2026-07-15

**Authors:** Ayman M. Atta, Gamal A. El-Mahdy, Hamad A. Al-Lohedan, Abdurrahman O. Ezzat

**Affiliations:** 1 Surfactants Research Chair, King Saud University, Chemistry Department, College of Science, P.O. Box-2455, Riyadh-11451, Saudi Arabia; gamalmah2000@yahoo.com (G.A.E.-M.); hlohedan@ksu.edu.sa (H.A.A.-L.); ao_ezzat@yahoo.com (A.O.E.); 2 Petroleum Application Department, Egyptian Petroleum Research Institute, Cairo 11727, Egypt; 3 Chemistry Department, Helwan University, Helwan, Cairo 11795, Egypt

The journal retracts the article titled “Synthesis and Application of Hybrid Polymer Composites Based on Silver Nanoparticles As Corrosion Protective for Line pipe Steel” [1], cited above.

Following publication, concerns were raised with the Editorial Office regarding the integrity of the data and figures presented in this publication [1].

In accordance with the journal’s standard procedures, the Editorial Office and Editorial Board conducted an investigation that identified indications of inappropriate image editing and duplication in panels presented in Figures 1–3, including material overlapping with previously published articles [2].

Although the authors cooperated with the investigation and provided supporting materials, the Editorial Board concluded that the concerns could not be satisfactorily resolved based on the available information. As a consequence, the Editorial Board lost confidence in the validity of the images and has decided to retract this publication in accordance with MDPI’s retraction policy.

This retraction was approved by the Editor-in-Chief of the journal *Molecules*.

The authors have been informed of this retraction, but did not provide a comment on this decision.
